# Rhizome Severing Increases Root Lifespan of *Leymus chinensis* in a Typical Steppe of Inner Mongolia

**DOI:** 10.1371/journal.pone.0012125

**Published:** 2010-08-12

**Authors:** Wenming Bai, Fen Xun, Yang Li, Wenhao Zhang, Linghao Li

**Affiliations:** 1 State Key Laboratory of Vegetation and Environmental Change, Institute of Botany, Chinese Academy of Sciences, Beijing, China; 2 Graduate School of Chinese Academy of Science, Yuquanlu, Beijing, China; University of Leipzig, Germany

## Abstract

**Background:**

Root lifespan is an important trait that determines plants' ability to acquire and conserve soil resources. There have been several studies investigating characteristics of root lifespan of both woody and herbaceous species. However, most of the studies have focused on non-clonal plants, and there have been little data on root lifespan for clonal plants that occur widely in temperate grasslands.

**Methodology/Principal Findings:**

We investigated the effects of rhizome severing on overall root lifespan of *Leymus chinensis*, a clonal, dominant grass species in the temperate steppe in northern China, in a 2-year field study using modified rhizotron technique. More specifically, we investigated the effects of rhizome severing on root lifespan of roots born in different seasons and distributed at different soil depths. Rhizome severing led to an increase in the overall root lifespan from 81 to 103 days. The increase in root lifespan exhibited spatial and temporal characteristics such that it increased lifespan for roots distributed in the top two soil layers and for roots born in summer and spring, but it had no effect on lifespan of roots in the deep soil layer and born in autumn. We also examined the effect of rhizome severing on carbohydrate and N contents in roots, and found that root carbohydrate and N contents were not affected by rhizome severing. Further, we found that root lifespan of *Stipa krylovii* and *Artemisia frigida*, two dominant, non-clonal species in the temperate steppe, was significantly longer (118 d) than that of *L. chinensis* (81 d), and this value became comparable to that of *L. chinensis* under rhizome severing (103 d).

**Conclusions/Significance:**

We found that root lifespan in dominant, clonal *L. chinensis* was shorter than for the dominant, non-clonal species of *S. krylovii* and *A. frigida*. There was a substantial increase in the root lifespan of *L. chinensis* in response to severing their rhizomes, and this increase in root lifespan exhibited temporal and spatial characteristics. These findings suggest that the presence of rhizomes is likely to account for the observed short lifespan of clonal plant species in the temperate steppe.

## Introduction

Fine roots represent the most important active carbon pool in terrestrial ecosystems, and root lifespan is an important parameter that regulates carbon and nutrient budgets in plants as well as in ecosystems [1]–[3]. Root lifespan also reflects the ability for plants to acquire and conserve soil resources in response to stressed environment [4], [5]. Root lifespan is highly variable, ranging from a few days to several years among different plant species [6]. A number of studies show that root lifespan is highly dependent on the size, species, and nutritional status of roots (e.g. root N concentration, allocation of carbohydrates from shoots to roots), being sensitive to changes in environmental factors such as soil resource availability [5]–[8].

Up to now, most studies on root lifespan have focused on woody plants, while there is little information on root lifespan of grasses in general, and clonal grasses in particular. For rhizomatous clonal grasses, rhizomes play an important role in adaptation to heterogeneous habitats by facilitating exchanges of nutrients, water and carbohydrates among clonal ramets [9]–[12]. Previous studies have shown that root lifespan of non-clonal grass species is sensitive to soil N availability [5], [13]. In this context, it is unknown whether the presence of rhizomes affects root lifespan via changing root N status. In addition to N, soluble carbon transfer from canopy to roots is also related to root lifespan [14], [15], such that decreased C allocation to roots reduces root lifespan [6]. For clonal species, a large portion of soluble carbon in roots is exchangeable between ramets via rhizomes [11], [16]. In addition, physiologically rhizomes are also “sinks” competing for photoassmilates with roots. It has been speculated that the presence of rhizomes in clonal plants may affect C contents in roots. Therefore it is expected that rhizomes may play an important role in regulating lifespan for roots of clonal plants. Moreover, root lifespan of non-clonal species depends on soil depth and the seasons that roots are born, and these characteristics are relatively independent of soil resource availability [4], [17], [18]. However, there has been limited report on the dependence of root lifespan on soil depths and seasons that roots are born in clonal species [19]. Given that clonal plants occur widely in grassland ecosystems, a better understanding of the role of matter exchanges via rhizomes between ramets in modulating root longevity of clonal plants is critical in elucidating mechanisms for plants to efficiently utilize soil resources and the strategies in their adaptation to unfavorable habitats [6].

Rhizome severing by plowing the pasture floor has been a commonly adopted practice in restoring and improving degraded grasslands dominated by rhizomatous clonal plant species in Eurasian grasslands. Although the effects of rhizomes severing on aboveground plant production and species diversity have been widely examined [11], [20], the potential effects of rhizome severing on root dynamics and longevity in clonal plants have not been reported in the literature. In the present study, we examined the effects of rhizome severing on the overall root lifespan of *L. chinensis* in a 2-year field study using improved digital photography and root-windows techniques. We also investigated responses of lifespan for roots born in different seasons and occurred at different soil depths to rhizome severing. Finally, we compared root lifespan of clonal species of *L. chinensis* with that of non-clonal, dominant species of *S. krylovii* and *A. frigida* under the identical conditions in the temperate steppe.

## Methods

### Study Site

The experiment was conducted in a temperate steppe (41°46'N, 115°51'E, 1215 m above sea level) in Duolun county, Inner Mongolia, China. The soil belongs to chestnut soil. Soil texture is sandy, and the mean soil pH value is about 6.8. This area is characterized by a semiarid continental monsoon climate, with a mean annual air temperature of 2.1°C. Mean annual precipitation is about 385.5 mm, while mean potential evaporation is around 1748.0 mm. Field observations were made in the permanent experimental enclosure of the Duolun Restoration Ecology Research Station of Chinese Academy of Sciences. Plant community was a monoculture of *L. chinensis,* which accounted for over 95% of the total aboveground biomass [16]. This species is a typical clonal plant with ramets, among which nutrients and water could be exchanged through rhizomes [20]. The majority of rhizomes are located at the top soil layer (0–10 cm) [16]. To compare root lifespan of *L. chinensis* with other species in the experimental area, plant communities with non-clonal, dominant species of *S. krylovii* and *A. frigida* were also chosen to investigate root dynamics. Plant growth starts in mid-April and senesces in late September.

### Experimental Design

Treatment was initiated in the autumn of 2003. An area of 15 ha was divided into ten 5×8 m^2^ plots, and the 10 plots were randomly laid out to be 2 rows and 5 columns with a 1-m buffer distance between any two plots. There were five replicates in both control and rhizome severing plots, respectively. To minimize the disturbance of soil surface, we severed rhizomes by ploughing to 15 cm soil depth by a flat shovel (0.3 mm in thickness) along parallel lines (20 cm apart) on 11 Sept. 2003.

### Installation of Glass-root Windows

Glass-root windows were used for root lifespan observations in the study. On the same date (11 Sept. 2003) when rhizome-severing treatment was made, one glass-root window was installed in each plot. The glass window being 0.4 cm thick, 40 cm in height and 50 cm in length was installed vertically into the soil. On each glass window, a 30×40 cm panel was divided into twelve 10×10 cm squares by carving the glass. A vertical soil profile of similar dimensions was dug in each plot. Glass window was installed tightly to the profile and fixed with one iron stick at each side, after which soil was backfilled as tightly. In order to minimize the effects of light on root growth, the upper edge of glass windows was inserted 1 cm under the soil surface and a piece of dark iron was covered on the top of the glass. All glass windows were placed perpendicularly with the direction of cutting lines. To minimize the effects of glass windows per se on root growth and soil regimes, a buffer time of about 7 months was maintained before observations started. In addition to *L. chinensis* community, we also studied root dynamics in a community that is dominated by *S. krylovii* and *A. frigida* (five plots of 3×4 m^2^) using the same method in the same experimental area following the protocols used for *L. chinensis* described above.

### Image Collection and Processing

Detailed protocols used for image collection and processing basically follow those described previously [19], [21]. Briefly, a digital camera was used to observe root growth. Observation began on May 11, 2004, and lasted till 12 September 2005 with a sampling interval of about 10 d following rhizome severing on 11 Sept. 2003. On each observation occasion, the temperately-backfilled soil of approx. 30 cm in thickness at the outside of glass windows was removed and the screen was cleaned as transparent as possible. One digital picture was taken for each of the twelve numbered squares. Removed soil was immediately refilled again after pictures were taken.

The software of Mapinfo professional (5.0) and a computer mouse were used to analyze the processes of appearance and disappearance of fine-roots. For the initially collected images, each root was assigned with an identification number and distinguished as living or dead based on color, and the same criterion was used throughout this study. For the following image sets, the tracings from the previous date were compared with the new images, allowing previously existing roots to be identified. Newly emerged roots were also identified and numbered. Roots that had disappeared at subsequent images were assumed to be dead and decomposed. Complete records were kept for all roots, even for those that were classified as dead.

### Analysis of Root Survival

Root lifespan was estimated as the date on which roots were observed as black or disappeared minus the date on which the roots were initially observed on the window. The date of root appearance or disappearance was estimated as the date midway of the sampling period because they might have occurred on any day during the approx. 10-d sampling interval between two consecutive observations [4], [17], [22].

We selected total of 2185 new roots germinated in spring (May 11–23), summer (July 19–30) and autumn (24 August-September 4) of 2004 to analyze the effects of root birth timing and rhizome severing on their survival rates and longevity. We calculated the overall mean and median root longevity through a survival curve using Kaplan-Meier method with SPSS (12.0) software, and compared the root survival rates by Log-rank test.

A stratified Cox proportional hazards regression was made to estimate the effects of treatment, soil depth and birth season on root lifespan as described by Gill *et al*. (2002). In the Cox model, the hazard for an individual root at time t is computed based upon the combination of an unspecified baseline (h_0_) function and an exponential function of k covariates:
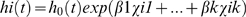



The Cox model was used to estimate *β* coefficient for each model covariate and test the null hypothesis of *β* = 0 with a chi-square statistic. A negative *β* coefficient indicates a decreased risk of mortality with an increase in the parameter. A risk ratio (*exp β*) being used to calculate the percent change in the risk of mortality with a one unit change in the covariate was calculated as well [23], [24].

### Effect of Rhizome Severing on Root Production

Protocols used for determination of root production in the present study were as described previously [19]. Briefly, root production for each sampling period was measured by summing the length of all new roots and adding the extension growth of all previously existing roots.

### Measurements of Soil Moisture and Nitrogen

Gravimetrical soil water contents at 0–10 cm, 10–20 cm and 20–30 cm soil depths were measured on the same day when root images were collected. Repeated measure ANOVA was used to detect differences in soil water content between treatments and between soil layers. Soil samples at the soil depths of 0–10 cm, 10–20 cm and 20–30 cm were taken in the spring, summer and autumn of 2004, respectively, and mineral N (NH_4_^+^-N, NO_3_^−^-N) concentrations were measured using a continuous-flow ion auto-analyzer (Scalar SANplus segmented flow analyzer, the Netherlands). Two-Way ANOVA and Duncan's Multiple-Range Test were conducted to compare the differences in soil NO_3_^−^-N and NH_4_^+^-N concentrations between treatments and soil depths for different seasons.

### Measurements of Plant Biomass and Nutrient Concentration

Maximum canopy biomass was determined in 2004 and 2005 by harvesting all plant materials in the quadrates. Five 1×1 m^2^ quadrats were randomly fixed for the control and rhizome-severing plots separately. Roots born in different seasons were sampled separately for measuring root nutrient concentrations, with the roots of the three soil layers being pooled together. Five replicates with each replicate containing three soil cores for both the control and rhizome severing were fixed for each season. Living roots taken using soil-sampling appliance (30 cm in length, 12.5 cm in diameter) in 2004 and 2005 after determination of maximum canopy biomass were washed and oven-dried to constant weight. Root N concentration was measured colorimetrically by the Kjeldahl acid-digestion method with an Alpkem auto-analyzer (Kjektec System 1026 distilling unit, Sweden) after extraction with sulfuric acid. Root soluble C concentration was measured by the anthrone method [25], [26] and a UV-VIS7500 spectrophotometer (Techcomp, Shanghai, China).

## Results

### Effects of Rhizome-Severing on Soil Resource Availability

**Figure 1:**
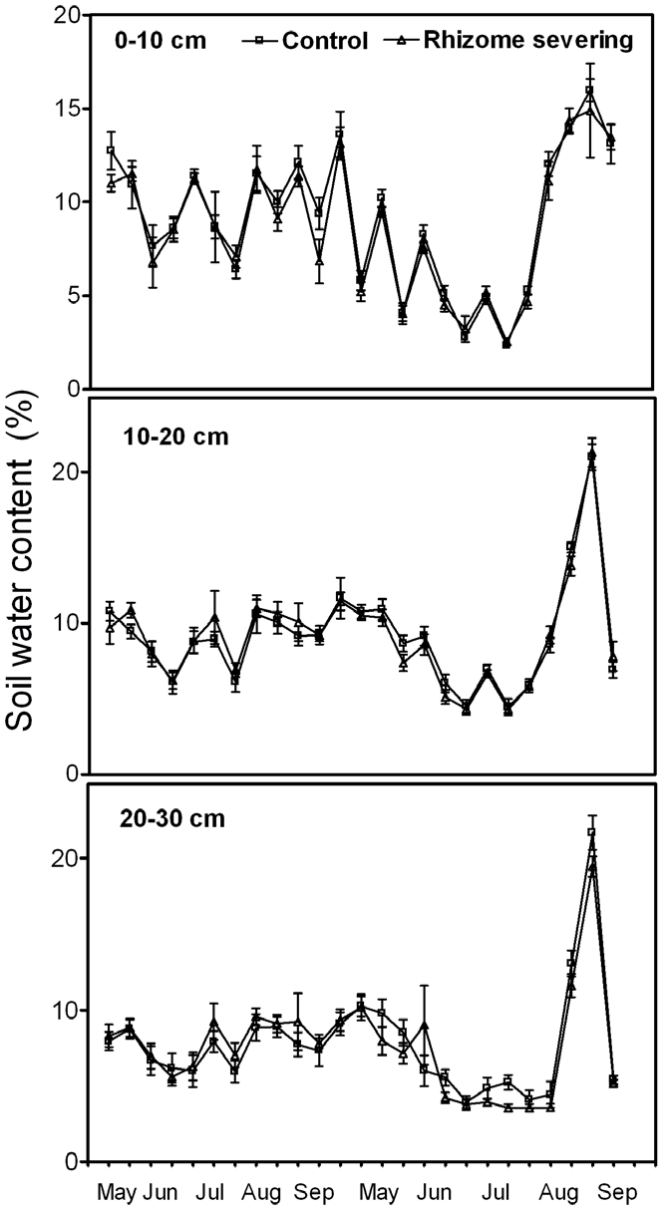
Seasonal fluctuations in soil water contents for the control and rhizome-severed plots at the soil depths of 0–10, 10–20 and 20–30 cm in the growing seasons of 2004 and 2005. Data are mean ± SE for five replicates.

There were substantial temporal changes in soil water contents for each soil layer for both control and rhizome-severed plots in the growing seasons of 2004 and 2005. Similar seasonal changes in soil water contents were found for different soil layers (Fig. 1). In addition, there were no significant differences in soil water contents between the control plots and rhizome-severed plots in both years, regardless of soil depth and season. Similar to soil water contents, there were no significant differences in soil NO_3_^−^-N concentrations between the control and rhizome-severed plots, regardless of soil depth and season (Table 1). Moreover, soil NH_4_^+^-N concentrations were not significantly different between control and rhizome-severed plots (data not shown).

**Table 1:** Soil NO_3_^−^-N concentrations (mg kg^−1^) at the soil depths of 0–10, 10–20 and 20–30 cm in spring (May 9), summer (July 19) and autumn (September 14) of 2004 for control and rhizome severing treatments.

Observation date	Soil depth (cm)	Control	Rhizome severing
9-May	0–10	5.50±1.07 a	4.27±0.68 a
	10–20	2.78±0.81 a	1.94±0.17 a
	20–30	1.65±0.26 a	2.22±0.32 a
19-July	0–10	9.48±1.47 a	9.89±0.71 a
	10–20	9.11±1.66 a	10.91±1.35 a
	20–30	8.32±1.45 a	9.78±0.71 a
14-September	0–10	3.69±0.63 a	4.57±1.25 a
	10–20	3.94±1.02 a	4.39±1.19 a
	20–30	5.20±0.91 a	4.07±0.73 a

Values shown in the table are the mean ± SE of five independent replicates. In each horizontal row, the values with the same superscript letters are not significantly different from each other at *P* = 0.05.

### Response of Root Lifespan to Rhizome Severing

**Figure 2:**
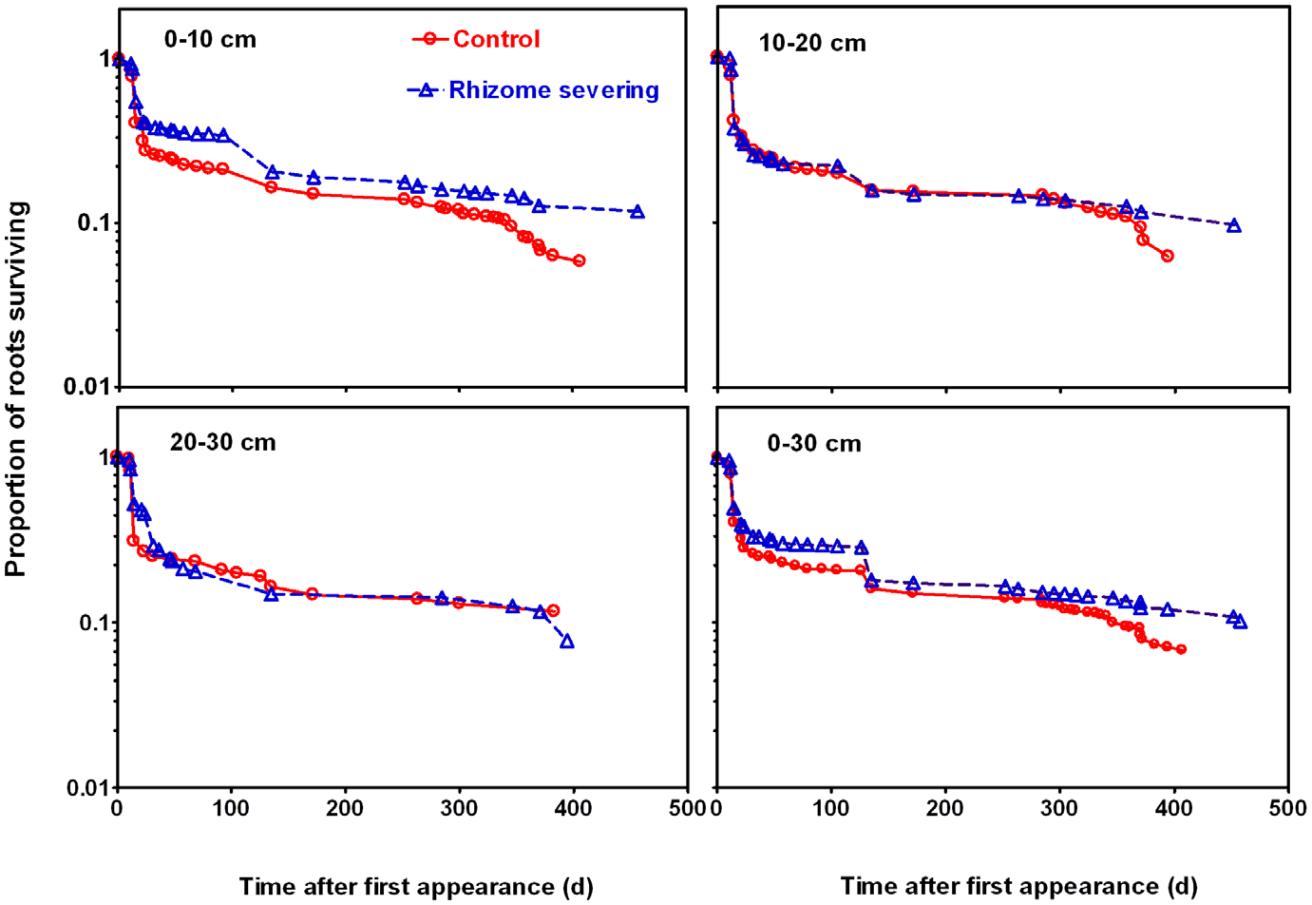
Root lifespan of *L. chinensis* at soil depths of 0–10 cm, 10–20 cm and 20–30 cm (mean ± standard error) and the overall lifespan of roots occurred in the three soil layers and in different seasons pooled as a whole in the control and rhizome severed plots. Data shown in the figure were based on a total number of 1018 and 1167 individual roots for control and rhizome severing respectively. Mean lifespan was estimated by the Kaplan–Meier survival analysis approach. Differences in lifespan between the control and rhizome severing were compared using Log-rank test. The values for each control and rhizome-serving pair with same superscript letters are not significantly different from each other at *P* = 0.05.

There was a significant increase (*P*<0.01) in the overall lifespan of roots in the three soil layers and in different seasons pooled as a whole in rhizome severed plots compared with root lifespan in control plots during the experimental period (Fig. 2). As far as soil depth is concerned, lifespan for roots distributed in the topmost soil layer increased significantly in response to rhizome severing, while no effects of rhizome severing on lifespan were found for those roots in the other two soil layers. The hazard analysis model revealed that rhizome severing decreased the overall root mortal risk of *L. chinensis* by 17.5% (*P*<0.001) (Table 2).

**Table 2:** Results analyzed by the Cox proportional hazards regression on roots of *L. chinensis* in relation to treatment, soil depth and time of root birth respectively.

Covariate	Parameter estimate	SE	Hazard Ratio	95% CI Lower Upper		*P*-value
TreatmentRef. = Control (CK)Rhizome severing (RS)	−0.192	0.025	0.825	0.786	0.867	<0.001
Soil depth (CK)Ref. = 0–10 cm10–20 cm20–30 cm	0.03−0.042	0.550.70	1.0030.959	0.9000.836	1.1171.100	0.9620.549
Soil depth (RS)Ref. = 0–10 cm10–20 cm20–30 cm	0.0730.022	0.0510.067	1.0761.023	0.9730.897	1.1891.165	0.1530.738
Root birth (CK)Ref. = SpringSummerAutumn	−0.518−0.415	0.040.05	0.5960.661	0.5390.598	0.6340.730	<0.001<0.001
Root birth (RS)Ref. = SpringSummerAutumn	−0.118−0.321	0.0670.049	0.8890.725	0.7780.658	1.0140.799	<0.001<0.001

Hazard ratios for different categorical covariates are the mortal risk relative to a reference level, given for each covariate in the left-hand column. A negative parameter indicates that increases in the covariate would result in decreases in the mortal risk, while a positive parameter indicates the opposite trend. Confidence intervals of 95% are upper and lower limits. *P*<0.05 is considered to be significant.

**Figure 3:**
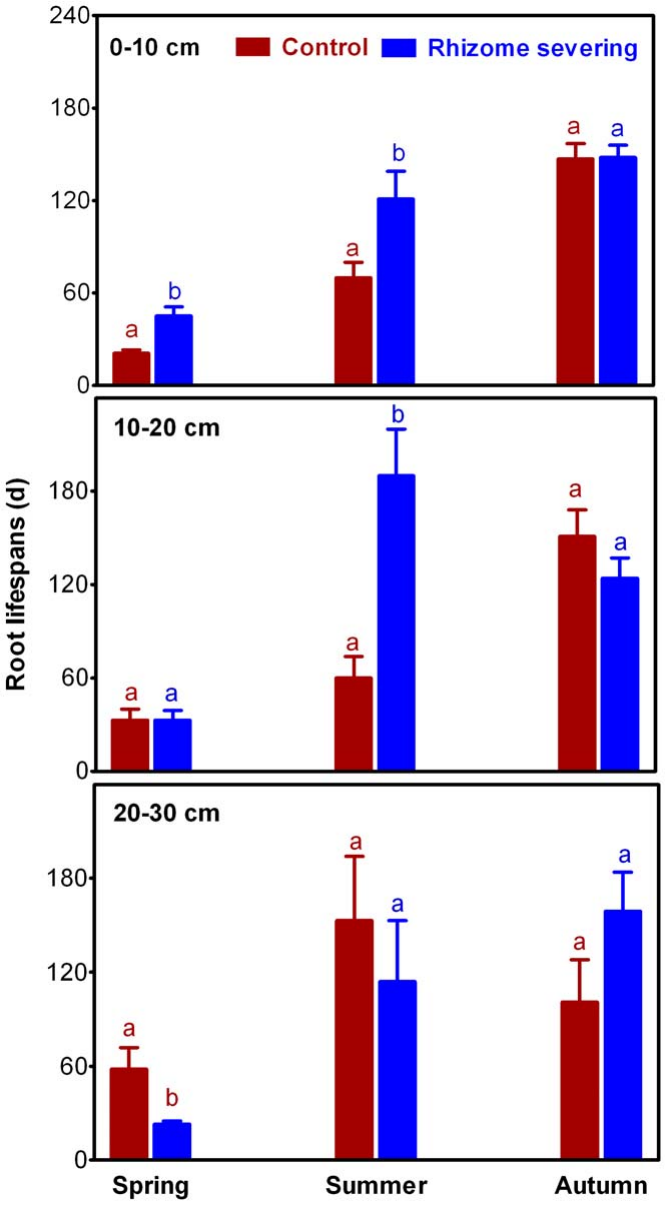
Lifespan of roots born in spring, summer and autumn in different soil depths in the control and rhizome-severed plots. Data were based on the total numbers of 997, 377 and 811 individual roots born in spring, summer and autumn, respectively. The Kaplan–Meier method was used to generate curves and to estimate mean lifespan. Survival differences between the timing of root birth were compared using Log-rank test.

Effects of rhizome severing on root lifespan differed significantly among roots born in different seasons. Lifespan for roots born in summer in the 0–30 soil layer pooled as a whole was increased from 73 to 142 d after rhizome severing, whereas lifespans for roots born in spring and autumn were not affected by rhizome severing (Fig 3). In addition, the seasonal effects of rhizome-serving on lifespan of roots born in different seasons differed with soil depth (Fig. 3). For example, rhizome severing increased lifespan for roots that were born in summer in the soil layers of 0–10 cm and 10–20 cm by 72% and 217%, respectively. In contrast, lifespan for the summer-born roots in the deepest soil layer (20–30 cm) was not affected by rhizome severing (Fig. 3). Lifespan for roots born in spring in the top and deepest soil layers was markedly increased and decreased by rhizome severing, respectively (Fig. 3). In contrast, there was no change in lifespan for spring-born roots in the soil layer of 10–20 cm in response to rhizome severing (Fig. 3). Unlike those roots born in spring and summer, rhizome severing had no effect on lifespan of autumn-born roots in all the three soil layers examined (Fig. 3). In addition, we found that lifespan for roots born in the three seasons followed the general pattern of autumn-born roots > summer-born roots >spring-born roots when the roots in the three soil layers pooled together. This trend of dependence of root lifespan on root-born seasons was not changed by rhizome severing (Fig. 3). The Cox proportional hazards regressions revealed that the mortal risk of roots born in summer and autumn was lower than that of roots born in spring for both control and rhizome severing plants (Table 2).

**Figure 4:**
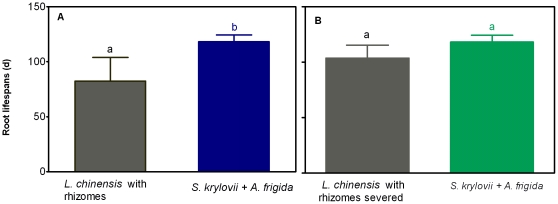
Comparison of overall root lifespan of clonal species of *L. chinensis* before and after severing rhizomes with non-clonal species of *S. krylovii* and *A. frigida.* Data are mean ± SE of four replicates. Data with different letters mean significant difference at *P*<0.05 levels.

To test whether root lifespan in clonal species is shorter than that of non-clonal species, we measured root lifespan of dominant, non-clonal species of *S. krylovii* and *A. frigida* using the same methodology. As shown in Figure 4A, the overall root lifespan of *S. krylovi*i and *A. frigida* was significantly larger (P<0.05) than that of *L. chinensis* with rhizomes not being severed. However, the root lifespan of *S. krylovii* and *A. frigida* was not significantly different (P = 0.154) from that of *L. chinensis* after rhizome severing (Fig. 4B).

### Effect of Rhizome Severing on Root Production

**Figure 5:**
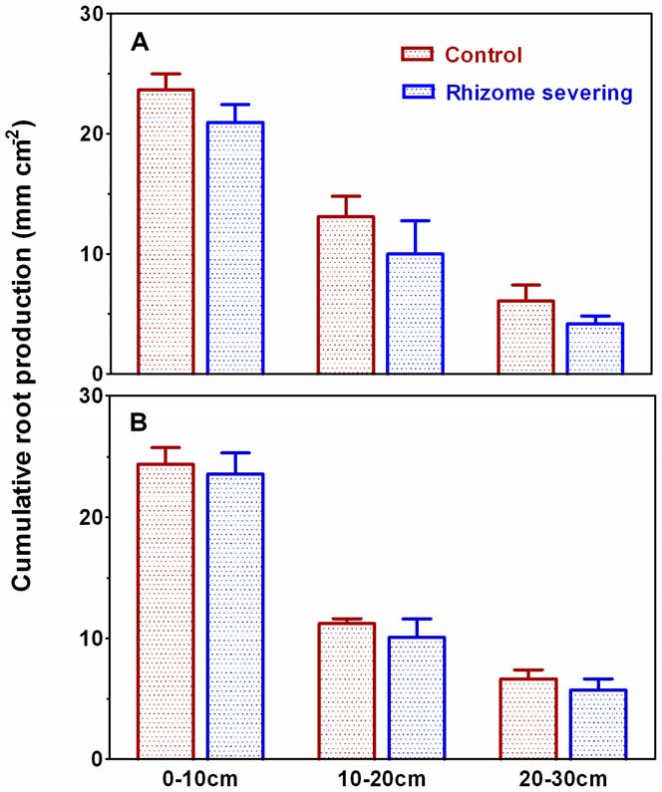
The cumulative root length production of *L. chinensis* in different soil depths in control and rhizome severing plots during growing seasons of 2004 (A) and 2005 (B). Data are mean ± SE for five replicates.

To examine whether rhizome severing-induced changes in root lifespan affected root production, we investigated the effect of rhizome severing on root production. There was no difference in cumulative root production between control and rhizome severing plots in 2004 and 2005, and the cumulative root production decreased with increasing soil layers in both control and rhizome severing plots (Fig. 5).

### Effects of Rhizome Severing on Plant Biomass and Contents of C and N

**Figure 6:**
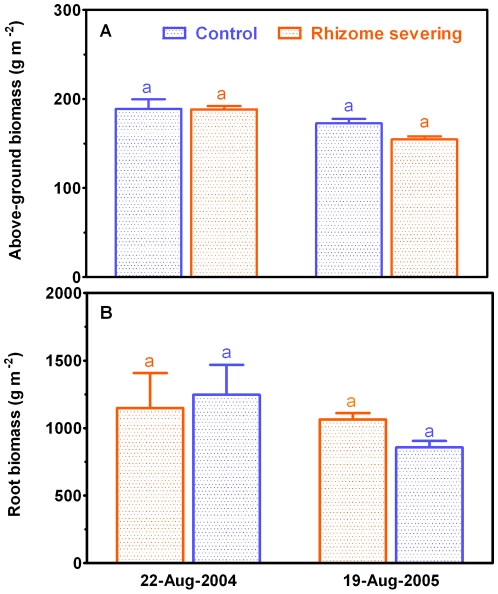
Above ground biomass and root biomass in control and rhizome-served plots in 2004 (A) and 2005 (B). The differences between control and rhizome-served plots were examined by student t-test, and the different letters given in the top of error bars indicate significant difference at *P*<0.05 levels. Data were mean ± SE of five replicates that were from five different plots.

**Figure 7:**
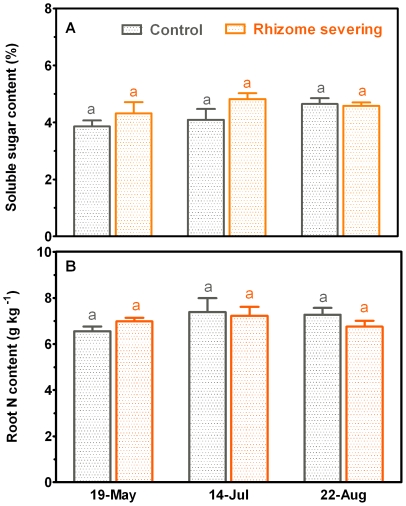
Root soluble C (A) and N (B) concentration in control and rhizome-served plots in 19-May, 14-Jul and 22-Aug of 2004. The differences between control and rhizome-served plots were examined by student t-test, and the different letters given in the top of error bars indicate significant difference at *P*<0.05 levels.

**Figure 8:**
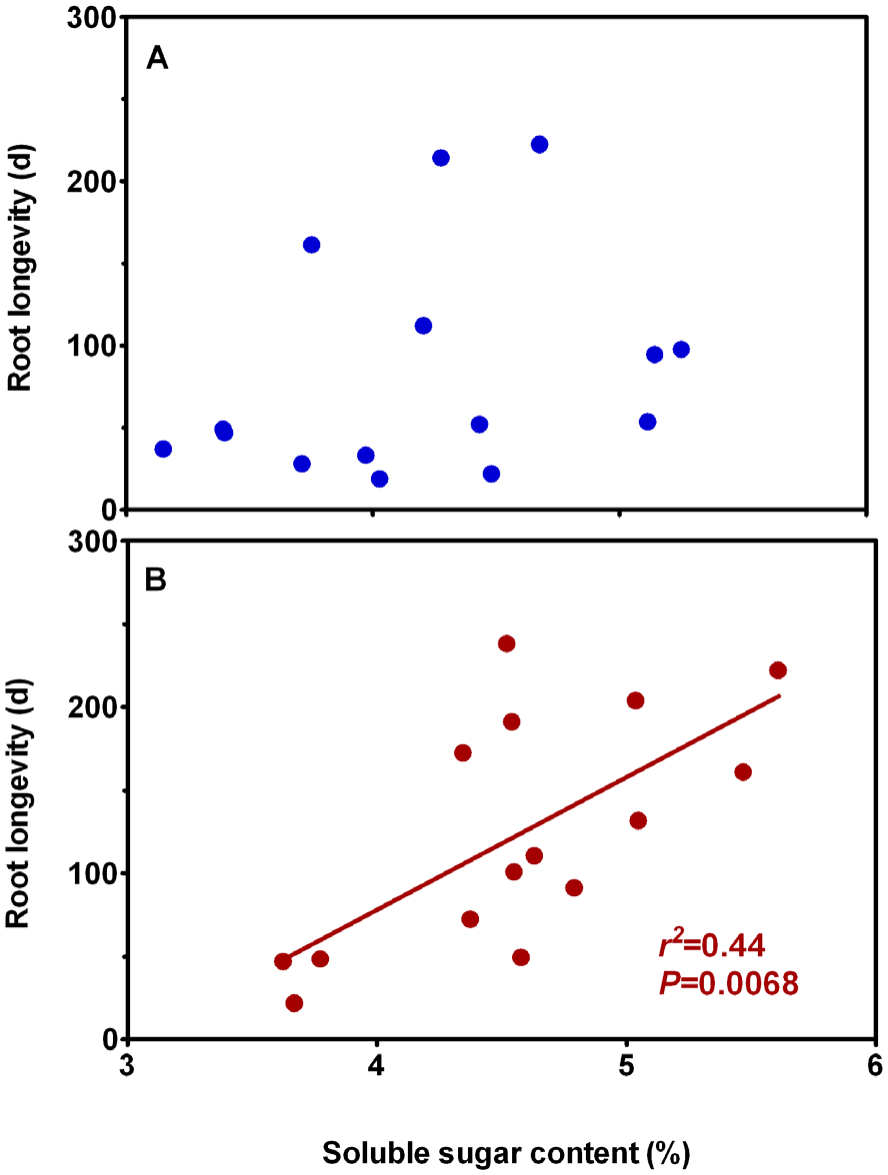
Regressions of mean root lifespan against root soluble C concentration for the control (A) and rhizome-severed (B) plots. Data for mean root lifespan were the mean values across the three soil layers.

Both above- and below-ground biomass of *L. chinensis* were not affected by rhizome severing across the 2-year experimental period (Fig. 6). It has been established that N and soluble carbon status in roots are closely associated with root lifespan [6]. To examine whether the changes in root lifespan in response to rhizome severing result from alterations in N and C status, we studied effect of rhizome severing on N and soluble C contents in roots of *L. chinensis*. There was an insignificant increase (*P* = 0.331) in soluble C contents in roots in response to rhizome severing (Fig. 7A). Rhizome severing had no effect on N contents in roots (Fig. 7B). When correlation between root lifespan and soluble C content was made across all the sampling seasons with roots in the three soil layers pooled together, no significant correlation was detected for the control plots (Fig. 8A). However, mean root lifespan became significantly correlated with soluble carbon concentration of roots in the rhizome-severed plots (Fig. 8B; *r^2^* = 0.44, *P* = 0.0068).

## Discussion

*Leymus chinensis* (Trin.) Tzvel, a perennial clonal grass, is widely distributed in the eastern region of the Eurasian steppe zone including the outer Baikal area of Russia, northern and eastern parts of the People's Republic of Mongolia, the Northeast China Plain, the Northern China Plain, and the Inner Mongolian Plateau of China, the total area of *L. chinensis* grasslands is about 420,000 km^2^ and 220,000 km^2^ in Asia and China, respectively [27]. Therefore, unraveling the characteristics of *L. chinensis* root lifespan is of significance for understanding C cycling in the Eurasian steppe. Our findings reveal that rhizome severing substantially increased the overall mean root lifespan and survival rate of *L. chinensis* (Fig. 2). More importantly, we found that the effects of rhizome severing on root lifespan displayed both temporal and spatial features such that lifespan for roots born in different seasons and distributed in different soil layers responded to rhizome severing differently. Our previous study has shown that N addition reduced lifespan of *L. chinensis* roots [19]. In the present study, we found that rhizome severing did not affect root N status in the present study, thus discounting the possibility that rhizome severing-induced changes in root lifespan is due to its effect on N status. As soil water contents in the control and rhizome-served plots did not differ (Fig. 1), it is unlikely that water availability in soils may be involved in the observed changes in root lifespan in response to rhizome severing. In addition to water and N, it has been reported that root lifespan is closely dependent on root soluble C content [14], [28], [29]. In contrast, lifespan for *L. chinensis* roots was not correlated with soluble C contents in roots in the control plots (Fig. 8A), while there was a weak positive correlation between the root lifespan and root soluble C contents in the rhizome severing plots (Fig. 8B). These findings imply that the changes in root soluble C may make small contribution to the increased root lifespan in rhizome-severing plots.

One important feature of *L. chinesis* roots is that its lifespan was much shorter compared to the reported lifespan values in the literature [5], [13], [23]. For example, Van Der Krift and Berendse (2002) reported that root lifespans of three herbaceous species are 280, 371 and 406 d for *Arrhenatherum elatius* L., *Molinia caerulea* L. and *Nardus stricta* L., respectively. We have speculated that the shorter root lifespan may be accounted for by the presence of extensive rhizomes which act as strong sinks to compete for photoassimilates, leading to lower soluble C in their roots [19]. In the present study, our findings that severing rhizome prolonged root lifespan provide evidence in support of the role of rhizomes in controlling root lifespan. It has been reported that soluble C contents in *L. chinensis* rhizomes are much higher than in roots [30]. This would mean that soluble C in rhizomes, but not soluble C in roots, may mainly be involved in determining lifespan of *L. chinensis* roots in the control plots. When the large pool of soluble C from rhizomes is no longer available after severing the rhizomes, root lifespan has to become dependent on root soluble C content. This explanation is also consistent with the observed significant correlation between root soluble C content and root lifespan under conditions of rhizome severing (Fig. 8B). In addition, both roots and rhizomes are “sinks” that compete for photoassimilates from “source”. Severing rhizomes could inevitably enhance capacity of roots to attract more photoassimilates. This may account for the observed marginal increase in soluble C in roots in the rhizome-severed plants (Fig. 7). The observations that root lifespan of dominant, non-clonal species of *S. krylovii* and *A. frigida* was significantly longer than that of clonal *L. chinensis* (Fig. 4A) and rhizome severing led to root lifespan of *L. chinensis* being comparable to that of *S. krylovii* and *A. frigida* (Fig. 4B) are in line with our arguments that presence of rhizomes contributes to shorter root lifespan in *L. chinensis*.

Our findings also show that the effects of rhizome severing on root lifespan of *L. chinensis* differed greatly among roots born in different seasons and occurred in different soil layers (Fig. 3, Table 2). The most significant effects of rhizome severing on root lifespan occurred in the topmost soil layer (cf. Fig. 3). In fact, the majority of *L. chinesis* roots is fines roots (less than 1 mm) and distributed in the top soil layer [20], and rhizomes also mainly occur in the top soil layer [16]. Therefore *L. chinesis* roots in the top soil layer are more closely linked to rhizomes, making the two organs directly compete for soil resources such as water and nutrients. It is expected that severing rhizomes would have more impacts on lifespan for roots in the top soil layer than those in deeper soil layers. In addition, severing rhizomes may also lead to more photoassimilates into those roots distributed in the top soil layers. It is worthy noting that severing rhizomes led to an increase in soluble C in roots that were distributed in 0–30 cm soil layer (Fig. 7). As we did not measure root soluble C contents in different soil layers, it is possible that severing rhizomes may mainly increase soluble C in roots that occur in the top soil layer exclusively. This would lead to a greater increase in soluble C in roots distributed in the top soil layer (0–10 cm) than the observed increase in soluble C contents in the roots distributed in the three soil layers as a whole. This explanation may also be used to account for the differential responses of roots in different soil layers to the rhizome severing. Another interesting finding in the present study is that lifespan for roots born in different seasons also responded to rhizome severing differently such that rhizome severing increased the mean root longevity of *L. chinensis* most significantly for roots born in summer. This could be explained by the fact that exchanges of carbohydrates between roots of ramets via rhizomes are most active and intensive in *L. chinensis* in summer, coincident with the maximum plant growth period [11]. Future studies are warranted to elucidate the mechanisms underlying the seasonal dependence of root lifespan on rhizomes. In addition, our results also showed that rhizome severing prolonged root lifespan of *L. chinensis,* suggesting that rhizome severing may lead to less C input into soil. However, rhizome severing did not affect cumulative root length production (Fig. 5), indicating that the amount of photoassimilate transported into roots is not affected. Taken together, these results demonstrate that rhizome severing may not directly lead to more C input in the soil.

In summary, our study revealed that the clonal grass of *L. chinensis* had a relative short overall root lifespan compared with root lifespan values for other plant species (including grass of *S. krylovii*) in the same temperate steppe. The root lifespan of *L. chinensis* exhibited substantial dependence on their rhizomes such that severing rhizome led to an increase in root lifespan values, and that this increase in root lifespan was related to seasons for roots born and distribution in soil depth. These results highlight that rhizomes may play a regulatory role in root lifespan of clonal grasses in temperate steppe in north China.
